# Exploring the phenolic profile, antibacterial, and antioxidant properties of walnut leaves (*Juglans regia* L.)

**DOI:** 10.1002/fsn3.3554

**Published:** 2023-07-11

**Authors:** Ammar B. Altemimi, Siba Mouid Al‐haliem, Zina T. Alkanan, Muthanna J. Mohammed, Mohammad Ali Hesarinejad, Mazin A. A. Najm, Aziz Bouymajane, Francesco Cacciola, Tarek Gamal Abedelmaksoud

**Affiliations:** ^1^ Department of Food Science, College of Agriculture University of Basrah Basrah Iraq; ^2^ Department of Dental Basic Sciences, College of Dentistry University of Mosul Mosul Iraq; ^3^ Department of Biology, College of Education for Pure Sciences University of Mosul Mosul Iraq; ^4^ Department of Food Processing Research Institute of Food Science and Technology Mashhad Iran; ^5^ Pharmaceutical chemistry department, College of Pharmacy Al‐Ayen University Thi‐Qar Iraq; ^6^ Team of Microbiology and Health, Laboratory of Chemistry‐Biology Applied to the Environment, Faculty of Sciences Moulay Ismail University Meknes Morocco; ^7^ Department of Biomedical, Dental, Morphological and Functional Imaging Sciences University of Messina Messina Italy; ^8^ Food Science Department, Faculty of Agriculture Cairo University Giza Egypt

**Keywords:** caffeic acid, hydroquinone, quercetin, walnut

## Abstract

The aim of this study was to identify phenolic compounds in walnut leaves from northern Iraq and evaluate their ability to act as antibacterial and antioxidant agents. Phenolic compounds were determined by reversed‐phase HPLC. Antibacterial activity was tested against various bacteria. Antioxidant properties were evaluated by various assays, including reducing power and DPPH radical scavenging activity. The HPLC profiles of walnut leaf fractions revealed quercetin, hydroquinone, 4‐hydroxybenzoic acid, and caffeic acid in three fractions. The inhibitory activity of DPPH was determined as 47.66, 32.41, and 51.90 μg/mL for fractions I, II, and III, respectively. For ferric reducing power activity, fraction II > fraction III > fraction I and the FRAP activity was observed as 64.43, 73.19, and 68.18 μg/mL for fractions I, II, and III, respectively. All extracted fractions had antibacterial properties against all bacterial strains tested. Observations showed that fraction I was able to produce similar zones of inhibition as streptomycin in most cases. These results suggest that the fractions of this plant extract are plausible natural antioxidants that could be used as prime candidates for the synthesis of antioxidant drugs that can be used for the treatment of many oxidative stress‐related diseases.

## Graphical abstract

Phenolic compounds of walnut leaves were determined by reversed‐phase HPLC. Antibacterial activity was tested against various bacteria. Antioxidant properties were evaluated by various assays, including reducing power and DPPH radical scavenging activity.

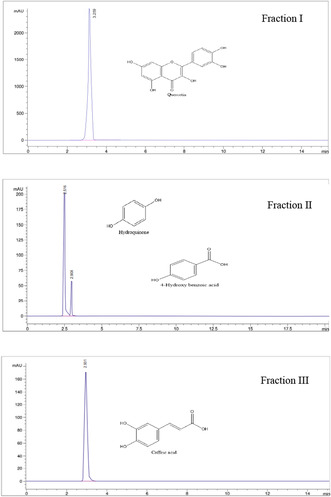

## 1 INTRODUCTION

The use of medicinal plants to cure ailments has been practiced for thousands of years across numerous countries. In previous centuries, natural remedies, particularly medicinal plants, were considered the mainstay of treatment. In recent years, there has been a growing inclination toward natural therapies in both developed and developing countries (Herpich et al., 2022). Medicinal plants are preferred over chemical drugs due to their lower cost and fewer side effects. Given the complications and harmful effects of chemical drugs, there is a serious consideration of using natural and herbal medicines. In fact, the use of herbal medicines has significantly increased in recent years (Tao et al., 2015). Furthermore, it is advised to seek necessary coordination and obtain scientific confirmation before using plants or their active components (Pai et al., 2023; Wu et al., 2023).

*Juglans regia* is one of the medicinal plants, commonly known as English walnut, and is a deciduous tree belonging to the family *Juglandaceae*. It is native to regions of central Asia, including parts of China, Iran, and Afghanistan, but is now widely cultivated throughout the world for its valuable wood and nutritious nuts. The tree can grow up to 25 m in height, with a broad crown of leaves and a strong, durable trunk (Gradziel et al., 2009; Jaiswal & Tailang, 2017; Shavvon et al., 2022; Spiegel‐Roy, 2019). Walnut leaves have long been used in traditional medicine due to their potential as a source of health‐promoting compounds. They are known to be effective in treating venous insufficiency, hemorrhoidal symptoms, diarrhea, and worm infestations, as well as possessing depurative and astringent properties (Delaviz et al., 2017; Gutiérrez Ortiz et al., 2018).

The therapeutic effects of *J. regia* leaves are attributed to their secondary metabolites, which include various compounds like phenolic acids, flavonoids, tocopherols, organic acids, triterpenic acids, terpenes, terpenoids, tetralone derivatives, megastigmane derivatives, hydroxy‐1,4‐naphthoquinone (*juglone*) derivatives, and others (Rather et al., 2012; Salimi et al., 2014; Santos et al., 2013; Schwindl & Kraus, 2017). Phenolic compounds are the predominant fraction in *J. regia* leaves, and they possess natural antioxidant properties. They are known for their radical scavenging activity, which helps to reduce oxidative stress—a leading cause of various disorders. Exogenous antioxidant sources like phenolic compounds play a crucial role in maintaining the oxidative stress balance, as the endogenous antioxidant system may not be sufficient (Carocho & Ferreira, 2013). Therefore, incorporating walnut leaf preparations (food and supplements) into our diet can act as a preventive medicine, providing numerous health benefits (Pires et al., 2018). *Juglans regia* also displays antimicrobial properties that enable it to prevent the growth or destroy microorganisms that can lead to infections (Elouafy et al., 2023). Research has demonstrated that several parts of the plant, including the leaves, bark, and nut, contain compounds that possess antimicrobial attributes and can effectively target a broad range of pathogens (Acquaviva et al., 2021; Barekat et al., 2022; Mateș et al., 2023; Żurek et al., 2022). The ability of *Juglans regia* to fight against microorganisms can be useful in various fields such as medicine, food preservation, and other industries where controlling the proliferation of microorganisms is crucial (Arslan et al., 2023; Bourais et al., 2022).

The extraction of bioactive compounds from walnut by‐products using various solvents is a key focus of research. This step is crucial in the production of products that are rich in phytochemicals. Utilizing this low‐cost technology to extract molecules from by‐products, such as walnut leaves, green husks, and membrane septum, is a suitable strategy for producing food additives and nutraceutical products (Jahanban‐Esfahlan et al., 2019; Popovici, 2013).

Evaluating the antioxidant and antimicrobial effects of walnut by‐products, including leaves, is crucial for discovering novel and alternative sources of antioxidants and nutraceuticals. This assessment has significant implications for their potential use in the food and cosmetics industries. The objective of this study was to identify the phenolic compounds present in walnut leaves grown in north region of Iraq and to assess their ability to act as both antimicrobial and antioxidant agents. To determine the phenolic compounds, reversed‐phase HPLC was employed. The antimicrobial activity was tested against different microorganisms including *Bacillus subtilis*, *Staphylococcus aureus*, *Pseudomonas aeruginosa*, and *Escherichia coli*. The antioxidant properties were evaluated through several tests, including reducing power and scavenging effects on DPPH radicals.

## 2 MATERIALS AND METHODS

### 2.1 Materials

During the flowering season of August and September 2022, the *Juglans regia* leaves were carefully collected from a specific location in Northern Iraq, chosen based on expert recommendations and previous research. The plant was grown in soil that was covered with sand and silt. The harvesting process was done under sterile conditions, with hand gloves and without causing any physical damage or exposing it to microbes. The Ministry of Agriculture in Baghdad confirmed the authenticity of the plant material. To avoid photodegradation of phenolic compounds, the plant materials were washed with sterile distilled water, dried in a dark room at about 25°C, and then dried further in a hot air oven at 36°C for 3 days. The dried plant parts were ground into a fine powder using a mortar and pestle, filtered through a sterile chiffon cloth, and stored at −20°C for later investigation.

### 2.2 Extraction of *Juglans regia* by Soxhlet

The powdered plant material (7 g) of *Juglans regia* was extracted using the Soxhlet extraction method with various solvents including methanol, hexane, ethyl acetate, and ethanol. All the solvents used were of analytical grade. The extraction process was carried out at a constant temperature of 60°C for 6 h and the complete process was replicated four times per solvent. After the extraction, the crude extract was evaporated using a rotary evaporator (Heidolph Instruments GmbH). The weight of all extracts was measured after the solvent evaporation and stored in an airtight container for further analysis (Elouafy et al., 2022).

### 2.3 Isolation of extract of walnut leaves by column chromatography

Silica gel column chromatography was utilized to extract the phytoconstituents from the *Juglans regia* extract. The column used for this purpose was a vertical glass one, made of borosilicate material, and measured 40 mm in width and 60 mm in length. Before packing, the column was rinsed with acetone and left to dry. A piece of glass wool was placed at the bottom of the column, followed by sea sand (with a particle size ranging from 50 to 70) to a height of 1 cm. Then, hexane was poured into the column, and 200 g of silica gel (with a mesh size of 60–120) was used as the packing material. A silica slurry was prepared with hexane and poured into the column until it reached 2/3 of its height. The slurry was then rinsed with solvent. Sea sand was added on top of the silica slurry to a height of 1 cm, and the sand particles were rinsed with the solvent. To isolate the fractions from the EAE, 20 g of it was mixed with the minimum amount of hexane and poured down the sides of the column, followed by rinsing with solvent. Sea sand was added on top of the EAE to a height of 1 cm, and the solvent level was maintained at 6 cm above the extract to prevent drying. The separation of fractions from EAE was achieved through a gradient elution method, varying the ratio of solvents (ethyl acetate, hexane, ethanol, and methanol). The flow rate was adjusted to 5 mL/min, and 40 mL of solvent was collected for each fraction. The concentrated fractions were analyzed using HPLC, and only the fractions that contained ethyl acetate and ethanol were identified. This was due to the difficulty in separating the hexane and methanol fractions, which resulted in an unrefined sample (Gini & Jeya Jothi, 2018).

### 2.4 Analysis of phenols by HPLC

RP‐HPLC (Agilent Technologies) was used to estimate the phenolic compounds in the pure *Juglans regia* leaf extract. They used a column called RP‐C18 end‐capped Lichrospher, which was 250 × 4.6 mm in size with 5‐μm particle size, and the column was heated to 40°C. The mobile phase was composed of a mixture of formic acid (0.1%) in double‐distilled water (A) and formic acid (0.1%) in acetonitrile (B). They used a gradient elution method, starting with 95% A for the first 15 min, followed by a decrease to 80% A for 5 min, then maintained at 70% A for 5 min, followed by a decrease to 10% A for 5 min, and then maintained at 10% A for 5 min, and finally increased to 95% A for the last 5 min. The injection amount was 5 μL, and the flow rate was 0.5 mL/min. They used a fraction collector with the RP‐HPLC system and after fractionation and concentration, each fraction was separately analyzed using a Nano‐ESI MS instrument (110–1500 *m*/*z*). They confirmed the identification of compounds by comparing retention times, fragmentation, and stepping methods using standard solutions and calibration curves (Raafat, 2018).

### 2.5 DPPH radical scavenging activity assay

The study measured the ability to scavenge the DPPH free radical using a method described in a previous study (Hatano et al., 1988). Different concentrations of the sample extracts (0.3 mL) were mixed with a methanolic solution containing DPPH radicals (6 × 10^−5^ mol/L). The mixture was shaken and left to stand in the dark until the absorption values stabilized. The decrease in absorption at 517 nm was continuously monitored to determine the reduction of the DPPH radical. The percentage of DPPH discoloration was calculated as the scavenging effect using the equation: % scavenging effect = [(*A*_DPPH_ × *A*_S_)/*A*_DPPH_] × 100, where *A*_S_ is the absorbance of the solution when the sample extract has been added at a particular level and *A*_DPPH_ is the absorbance of the DPPH solution. BHA was used as reference compounds (Oliveira et al., 2008).

### 2.6 Reducing power assay

The study measured the reducing power using a procedure described in previous studies (Ferreira et al., 2007; Oyaizu, 1986). Different concentrations of sample extracts (2.5 mL) were mixed with 2.5 mL of 200 mmol/L sodium phosphate buffer (pH 6.6) and 2.5 mL of 1% potassium ferricyanide. The mixture was then incubated at 50°C for 20 min. After incubation, 2.5 mL of 10% trichloroacetic acid (w/v) was added, and the mixture was centrifuged at 1000 rpm in a refrigerated centrifuge (Centorion K24OR‐2003) for 8 min. The upper layer (5 mL) was mixed with 5 mL of deionized water and 1 mL of 0.1% ferric chloride, and the absorbance was measured at 700 nm using a spectrophotometer. The study determined the extract concentration that provides 0.5 of absorbance (EC50) from the graph of absorbance registered at 700 nm against the correspondent extract concentration. BHA was as reference compounds.

### 2.7 Antimicrobial activity

Different strains of bacteria, such as *Bacillus subtilis*, *Staphylococcus aureus*, *Escherichia coli*, and *Pseudomonas aeruginosa*, were subjected to testing using extracts obtained from the leaves of *Juglans regia*. These microorganisms were obtained from the Biology Department of the University of Basrah in Iraq and were kept in storage on LB agar at a temperature of 4°C. The LB agar was composed of tryptone (1% w/v), yeast extract (0.5% w/v), NaCl (1% w/v), and agar (2% w/v), and the bacteria were regularly subcultured at a temperature of 37°C. An adaptation of the agar streak dilution method based on radial diffusion, as described by Sousa et al. (2006), was utilized to screen the antimicrobial activity against both Gram‐positive and Gram‐negative bacteria and determine the minimal inhibitory concentration (MIC) values. The microorganisms were suspended and mixed with 0.8% (w/v) molten agar to achieve a concentration of approximately 10^6^ CFU/mL. An 8‐mL volume of this mixture was spread evenly onto LB medium plates to form a lawn. Antimicrobial samples were tested by placing 85 μL in a 3 mm deep, 5 mm diameter hole at the center of the solid medium. The MIC was defined as the lowest concentration of the tested sample (ranging from 5 to 100 mg/mL) capable of inhibiting bacterial growth after 24 h at 37°C. The inhibition zone diameters were measured using a ruler with 0.5 mm accuracy, and the average radius of the inhibition zone in mm was calculated from three measurements on three different plates. Control plates inoculated with each sensitive indicator microorganism were also prepared (Fernández‐Agulló et al., 2013).

### 2.8 Statistical analysis

The complete experiments were implemented with three replications (*n* = 3), and all datasets obtained were statistically analyzed using statistical package for the social sciences (SPSS) software and the efficacy of plant extract fractions was analyzed using variance (ANOVA). The significant difference and the mean comparison were executed using Tukey's test with STATISTICA 13 (*α* = .05). Finally, the graph plots and results were analyzed using Microsoft Excel (2007) and BioStat software.

## 3 RESULTS AND DISCUSSION

### 3.1 Phenolic compound estimation

Fractions obtained from silica gel column chromatography of walnut (*Juglans regia* L.) leaf extracts were analyzed for the detection of various phytochemical compounds. For this purpose, the HPLC profiles of walnut (*Juglans regia* L.) leaf fractions were analyzed. Following extraction, three fractions were collected. Fraction I was associated with ethyl acetate extraction, while fractions II and III were associated with ethanol extraction. The identified phenolic compounds present in each fraction are shown in Table 1 with peaks having different retention times.

**TABLE 1:** Phenolic compounds detected in *Juglans regia* leaves with the different fractions by HPLC analysis.

Fractions	Number of peaks	Retention time (min)	Concentration (ppm)^c^	Identified compounds
I^a^	1	3.259	22.43 ± 2.76	Quercetin
II^b^	1	2.516	19.06 ± 1.98	Hydroquinone
	2	2.908	6.39 ± 1.04	4‐Hydroxybenzoic acid
III^b^	1	2.931	21.73 ± 2.73	Caffeic acid

^a^ Identified fractions from ethyl acetate extraction.

^b^ Identified fractions from ethanol extraction.

^c^ The values indicate the mean ± standard deviation (*n* = 3).

**FIGURE 1:**
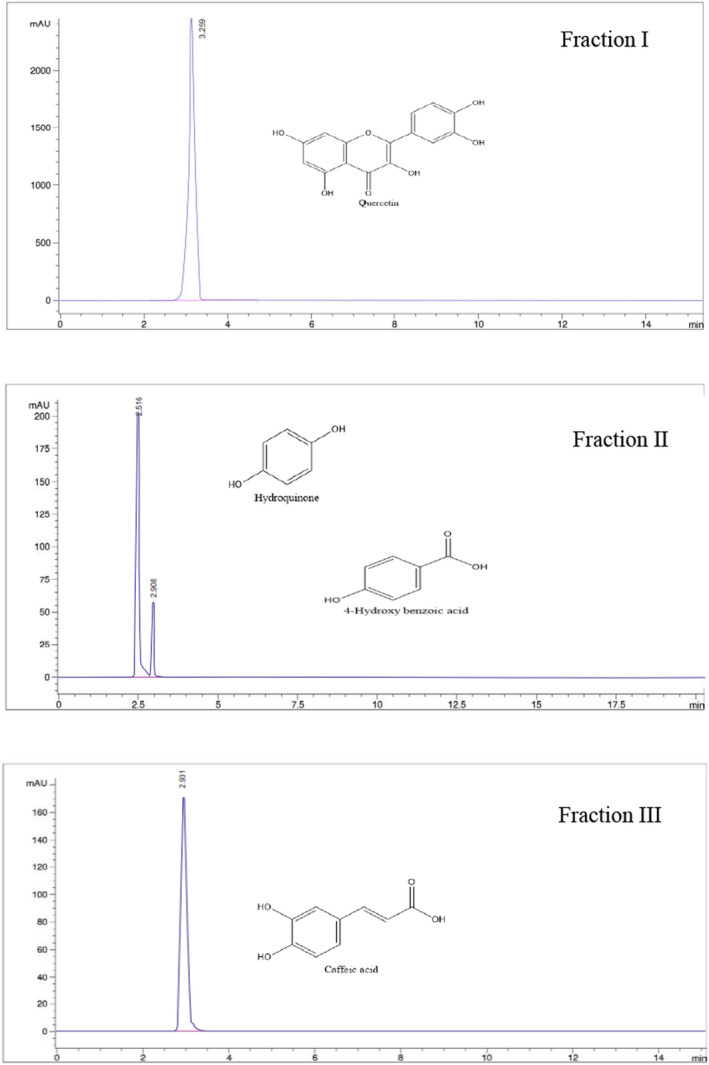
HPLC chromatograms of isolated fractions.

The active fractions obtained were as follows: The retention time of 3.259 min with a concentration of 22.43 ± 2.76 ppm formed fraction I; the retention times of 2.516 and 2.908 min with concentrations of 19.06 ± 1.98 and 6.39 ± 1.04, respectively, formed fraction II; the retention time of 2.931 min with a concentration of 21.73 ± 2.73 formed fraction III. In other words, one prominent peak was found in the HPLC chromatographic profile of Fraction I (Figure 1), which was recognized as quercetin based on its standard. Two main peaks were observed in Fraction II, which were recognized as hydroquinone and 4‐hydroxybenzoic acid (Figure 1). Caffeic acid was also found in Fraction III (Figure 1).

The results showed that the specific compound isolated in the phenolic compounds in walnut (*Juglans regia* L.) leaves extracted with ethyl acetate solvent was quercetin (Fraction I). Also, hydroquinone, 4‐hydroxybenzoic acid, and caffeic acid were introduced as specific compounds in ethanol solvent extraction (Fractions II and III; Figure 1). The results stated that the change in the polarity of the solvent and its solubility characteristics have led to the isolation of certain compounds. These were also consistent with the observations of Gawlik‐Dziki et al. (2014), who used the same plant material. In addition, various hydroxycinnamic acids and flavonoids (quercetin derivatives) of different walnut parts were studied by other researchers (Amaral et al., 2005).

### 3.2 Estimation of the antioxidant activity by DPPH method

Walnut (*Juglans regia* L.) leaves contain a variety of polyphenolic compounds that have antioxidant properties via diverse methods. In the present study, the antioxidant activity of fractions of walnut (*Juglans regia* L.) leaves was determined by the 2,2‐diphenyl‐1‐picrylhydrazyl (DPPH) radical scavenging assay (Table 2). This method has been frequently considered to evaluate the antioxidant activities of food materials (Koocheki et al., 2022).

**TABLE 2:** DPPH activity of isolated fractions.

Concentration (μg/mL)	Standard	Fraction I	Fraction II	Fraction III
2.5	41.06 ± 2.26^a^	30.11 ± 3.12^a^	32.04 ± 3.16^a^	29.11 ± 2.01^a^
5	46.14 ± 2.17^b^	35.05 ± 2.61^b^	37.36 ± 2.31^b^	32.42 ± 2.74^b^
20	58.21 ± 3.26^c^	41.13 ± 3.18^c^	49.11 ± 2.92^c^	39.82 ± 2.31^c^
40	69.74 ± 2.91^d^	49.33 ± 2.61^d^	52.32 ± 3.71^d^	44.32 ± 2.52^d^
80	84.29 ± 3.72^e^	52.11 ± 4.16^e^	59.11 ± 2.17^e^	50.81 ± 3.12^e^
160	98.35 ± 3.81^f^	66.44 ± 3.71^f^	70.45 ± 3.92^f^	58.32 ± 3.18^f^
IC50	10.73	47.66	32.41	51.90

*Note*: For a given proportion, means with the same superscript letters are not statistically different (*p* > .05).

Some researchers have reported the antioxidant activity of walnut leaves (Fernández‐Agulló et al., 2019; Pereira et al., 2008). In our research, all fractions showed relatively high antioxidant activity. The results revealed that with increasing the fraction concentration, the free radical scavenging power increased.

The free radical scavenging DPPH indicated the lowest calculated half‐maximal inhibitory concentration (IC50) value, which corresponds to the highest antioxidant potential. The calculated half‐maximal inhibitory concentration (IC50) value was determined as 47.66, 32.41, and 51.90 μg/mL for fractions I, II, and III, respectively and it was higher compared to BHA, used as a positive control (10.73 μg/mL). The highest antioxidant potential was observed for fraction II (IC50 = 32.41 μg/mL), whereas the lowest was observed (IC50 = 51.90 μg/mL) for fraction III (Table 2). Therefore, it can be stated that: antioxidant activity of hydroquinone and 4‐hydroxybenzoic acid > quercetin > caffeic acid. These values were similar to the results of other researchers who reported the antioxidant activity of *J. regia* (Tabaraki & Rastgoo, 2014; Zhang, 2015; Żurek et al., 2022). The acquired results were also lower when compared to earlier studies examining the antioxidant activity of leaves, nuts, and walnut shells evaluated in the DPPH test (Pereira et al., 2007, 2008). Natural antioxidants' primary function is to scavenge free radicals before they may trigger chain reactions in lipid‐rich matrices found in food, cosmetics, and pharmaceutical preparations, or in cell membranes (Alshahrani et al., 2022; Koşar et al., 2005).

### 3.3 Estimation of the antioxidant activity by ferric reducing power

The Ferric reducing power can be an important sign of antioxidant activity and is usually assessed by measuring the conversion of Fe^3+^ to Fe^2+^ in the presence of antioxidants. The antioxidant activity of *Juglans regia* leaves is primarily attributed to their high phenolic content. Different phenolic compounds have different antioxidant activities, which are most likely caused by the quantity of hydroxyl groups in the aromatic ring (Banc et al., 2023; Zhang et al., 2009). Quercetin exhibits antioxidant activity and acts as a radical scavenger (Cui et al., 2022; Pei et al., 2017). Caffeic acid is involved in the prevention of acute neuroinflammation (Castro et al., 2023; Mallik et al., 2016). Additionally, analgesic and anti‐inflammatory effects of hydroxybenzoic acid have been reported (Khan et al., 2016). As can be seen in Table 3, for FRAP activity, fraction I has lower antioxidant activity (64.43 ± 3.16 at 160 μg/mL) than the fractions in this study. Only fraction II, consisting of hydroquinone and 4‐hydroxybenzoic acid, showed a higher FRAP value than the other fractions (73.19 ± 3.41 at 160 μg/mL). Fraction III also showed good antioxidant activity. Previous studies have shown that caffeic acid has related biological activities such as antioxidant and anti‐inflammatory (Shiozawa et al., 2018). The relevant walnut matrix FRAP activity data ranged from 418.92 μmol Fe^2+^/g to 1067.94 μM Fe^2+^/g walnut leaf (Shah et al., 2018) and 454 μmol Fe^2+^/g walnut (Chen & Blumberg, 2008). Of course, an objective comparison of the results is unlikely, as no information was found on the FRAP activity of different fractions of walnut leaf extract.

**TABLE 3:** Ferric reducing power of isolated fractions.

Concentration (μg/mL)	Standard	Fraction I	Fraction II	Fraction III
2.5	44.32 ± 2.03^a^	26.22 ± 2.19^a^	30.02 ± 3.05^a^	28.29 ± 2.21^a^
5	50.18 ± 3.07^b^	30.21 ± 3.03^b^	35.51 ± 2.14^b^	31.41 ± 2.71^b^
20	59.84 ± 2.14^c^	35.91 ± 3.82^c^	41.27 ± 2.39^c^	38.42 ± 2.41^c^
40	70.11 ± 3.04^d^	44.92 ± 2.13^d^	48.54 ± 2.93^d^	47.21 ± 2.14^d^
80	87.22 ± 2.82^e^	55.81 ± 2.49^e^	63.69 ± 3.37^e^	59.81 ± 2.23^e^
160	98.51 ± 2.71^f^	64.43 ± 3.16^f^	73.19 ± 3.41^f^	68.18 ± 2.71^f^

*Note*: For a given proportion, means with the same superscript letters are not statistically different (*p* > .05).

### 3.4 Evaluation of the antibacterial activity

The antibacterial properties of walnut leaf fractions were also studied. Five Gram‐positive and Gram‐negative bacteria (*Bacillus subtilis*, *Staphylococcus aureus*, *Escherichia coli*, and *Pseudomonas aeruginosa*) were used. The antibacterial activity results of the extract and positive control are shown in Table 4. As shown in Table 4, the extract fractions possessed antibacterial properties against all tested bacterial strains. According to the zone of inhibition, all types of bacteria were not susceptible to the fractions extracted at 0.5 μg/mL. All studied bacterial types showed inhibition zones at concentrations higher than 2 μg/mL for fraction II, while this concentration was higher and equal to 4 μg/mL for fractions I and III. The antibacterial activities of the fractions at a concentration of 8 μg/mL ranged from 10 to 20 mm. A maximum zone of inhibition was observed in fraction I (20 mm), and a minimum zone of inhibition was achieved by fractions II and III (10 mm) against *P. aeruginosa*. As can be seen in Table 4, Streptomycin gave zones of inhibition of 21, 18, 20, and 16 mm against *B. subtilis*, *S. aureus*, *P. aeruginosa*, and *E. coli*, respectively. Fraction I at a concentration of 8 μg/mL showed corresponding zones of inhibition of 16 ± 1.25, 18 ± 1.27, 20 ± 1.28, and 16 ± 1.34 against the above bacteria, respectively. This observation indicated that fraction I could in most cases produce zones of inhibition similar to those of streptomycin.

**TABLE 4:** Antimicrobial activity of fractions I–III.

	Concentration (μg/mL)	Zone of inhibition (mm)			
		*B. subtilis*	*S. aureus*	*P. aeruginosa*	*E. coli*
Fraction I	0.5	0	0	0	0
	1	5 ± 1.09^b^	0	0	0
	2	8 ± 1.03^b^	10 ± 1.08^b^	0	8 ± 1.04^b^
	4	12 ± 1.04^c^	15 ± 1.44^c^	16 ± 1.11^b^	12 ± 1.17^c^
	8	16 ± 1.25^d^	18 ± 1.27^d^	20 ± 1.28^c^	16 ± 1.34^d^
Fraction II	0.5	0	0	0	0
	1	8 ± 0.71^a^	8 ± 1.03^a^	0	0
	2	10 ± 0.91^b^	10 ± 1.08^b^	6 ± 0.92^a^	8 ± 1.11^b^
	4	14 ± 1.08^c^	12 ± 1.13^c^	9 ± 1.07^b^	10 ± 1.41^c^
	8	16 ± 1.29^d^	14 ± 1.21^d^	10 ± 1.22^c^	12 ± 1.32^d^
Fraction III	0.5	0	0	0	0
	1	8 ± 0.36^a^	5 ± 0.61^a^	0	0
	2	10 ± 1.02^b^	8 ± 1.01^b^	0	0
	4	12 ± 1.06^c^	10 ± 1.03^c^	8 ± 1.11^c^	10 ± 1.64^c^
	8	15 ± 1.51^d^	12 ± 1.31^d^	10 ± 1.74^d^	12 ± 1.72^d^
Control	Streptomycin	21	18	20	16

*Note*: For a given proportion, means with the same superscript letters are not statistically different (*p* > .05); *B. subtilis*: *Bacillus subtilis*; *S. aureus*: *Staphylococcus aureus*; *P. aeruginosa*: *Pseudomonas aeruginosa*; *E. coli*: *Escherichia coli*.

Oliveira et al. (2008) previously assessed the green husks of walnuts' antibacterial activity. The antimicrobial activity of walnut septum was also investigated (Rusu et al., 2020). Keskin et al. (2012) used the disk diffusion method to assess the activity of the aqueous extracts and obtained higher zones of inhibition against *Pseudomonas fluorescens* (15 mm) and lower for *B. subtilis* and *P. aeruginosa* (8 mm). On the other hand, Sharma et al. (2013) examined the antibacterial properties of extracts made from green walnut husks using ethanol, ethyl acetate, and water. They reported that the ethanol extract had higher inhibitory diameters against the bacteria tested (*E. coli*, *Klebsiella pneumoniae*, *Staphylococcus aureus*) and the ethyl acetate extract was the most effective against *Bacillus subtilis*. To date, the antimicrobial activity of fractions of walnut leaf extracts has not been evaluated. It should also be noted that no statistical correlation was found between antimicrobial activity and chemical profile.

## 4 CONCLUSION

In conclusion, the findings of this study fill a gap in scientific knowledge about fractions extracted from *J. regia* leaves. This study confirms that walnut leaves (*Juglans regia* L.) are a valuable source of quercetin derivatives, hydroquinone, 4‐hydroxybenzoic acid, and caffeic acid. This is reflected in its high antioxidant capacity. In addition, the fractions of the extract from the leaves of *J. regia* have shown antibacterial activity. The observations showed that some of its fractions were able to produce zones of inhibition similar to streptomycin in most cases. According to these findings, it can be concluded that fractions of this plant extract are plausible natural antioxidants that could be exploited as a lead contender for synthesizing antioxidant medicines for the treatment of numerous oxidative stress‐related disorders.

## AUTHOR CONTRIBUTIONS

**Ammar B. Altemimi:** Conceptualization (equal); methodology (equal); writing – original draft (equal). **Siba Mouid Al‐haliem:** Data curation (equal); formal analysis (equal); writing – original draft (equal). **Zina T. Alkanan:** Data curation (equal); software (equal); visualization (equal); writing – original draft (equal). **Muthanna J. Mohammed:** Writing – original draft (equal). **Mohammad Ali Hesarinejad:** Conceptualization (equal); methodology (equal); writing – original draft (equal); writing – review and editing (equal). **Mazin A. A. Najm:** Software (equal); writing – review and editing (equal). **Aziz Bouimayane:** Writing – review and editing (equal). **Francesco Cacciola:** Writing – review and editing (equal). **Tarek Gamal Abedelmaksoud:** Conceptualization (equal); methodology (equal); writing – review and editing (equal).

## FUNDING INFORMATION

This research did not receive any specific grant from funding agencies in the public, commercial, or not‐for‐profit sectors.

## CONFLICT OF INTEREST STATEMENT

The authors declare that they have no known competing financial interests or personal relationships that could have appeared to influence the work reported in this paper.

## ETHICS APPROVAL AND CONSENT TO PARTICIPATE

This article does not contain any studies with human or animal subjects.

## CONSENT FOR PUBLICATION

All authors have read and agreed to the published version of the manuscript. All authors read and approved the final manuscript.

## Data Availability

All data generated or analyzed during this study are included in this published article.
